# Symptoms of COVID-19 Confirmed Cases Presenting to Emergency Department in A Tertiary Care Centre: A Descriptive Cross-sectional Study

**DOI:** 10.31729/jnma.5519

**Published:** 2020-12-31

**Authors:** Sumana Bajracharya, Ashis Shrestha, Bibek Rajbhandari

**Affiliations:** 1 Department of General Practice and Emergency Medicine, Patan Academy of Health Sciences, Lalitpur, Nepal; 2 Department of General Practice and Emergency Medicine, Nepal Police Hospital, Kathmandu, Nepal

**Keywords:** *clinical profile*, *comorbidity*, *COVID-19*

## Abstract

**Introduction:**

Clinical presentation of the patient with COVID-19 in emergency department is very important. The proper assessment of the symptom allows correct intervention. So, this study is conducted specifically to find out the clinical spectrum of the patient on presentation to emergency department.

**Methods:**

This was a descriptive cross-sectional study. A retrospective analysis of patient record was done. There were 258 COVID-19 positive cases admission from 13th April to 13th August 2020. Out of these cases 57 cases were excluded as they did not have respiratory symptoms but were admitted for other medical conditions. So, 201 symptomatic patients were analyzed in this study. Symptoms of all patient with the confirmed diagnosis of COVID-19 admitted from emergency department was analyzed. Data entry was done in excel sheet and presenting symptoms of COVID-19 positive patients were described along with their comorbid conditions.

**Results:**

Two hundred and one symptomatic patients were analyzed in this study. Mean age of this study population was 37.9 years (median 37) with minimum age of 2 months and maximum age of 83 years. There were 114 (56.7%) male and 87 (43.3%) female; 109 (54.2%) patients were from outside the and 92 (45.8%) were from inside of Kathmandu Valley. The most common presenting symptom was fever 131 (65.2%) and cardiovascular condition including hypertension was the most common comorbid condition.

**Conclusions:**

Fever was the most common symptoms of the patient presenting to COVID19 emergency of our hospital. Moreover, fever needs to be analyzed carefully in terms of its onset total duration and associated cough and underlying comorbid condition.

## INTRODUCTION

As of 10 July 2020, there are 16649 positive cases of Corona virus disease 2019 (COVID-19) in Nepal with most of the population being in the age group of 21 to 30 years.^1^ As Patan hospital has been declared as one of the centers for admitting patients with confirmed cases of Novel Corona virus, we are also getting increasing number of patient daily.

There is limited data on clinical profile of patient published from Nepal. A publication of four cases during initial days of COVID-19 showed fever and breathing difficulty as common symptoms in those patients.^2^

So, this study was conducted to find out specifically, the clinical spectrum of the patient at the time of presentation to emergency department in a tertiary care center.

## METHODS

The descriptive cross-sectional study was conducted at the Emergency Department of Patan Hospital in the month July 2020. Ethical approval was taken from Institutional Review Committee- Patan Academy of Health Sciences (Ref no. drs 2008251435). All symptomatic COVID-19 patients who were admitted in this hospital through COVID-19 emergency from April to August 2020 were reviewed retrospectively. Presenting symptoms were recorded in admission information sheet^3^ provided by Nepal government.

Sample size was calculated by using the formula given below:

Sample Size (n) = Z^2^ pq/e^2^

where,

Z (level of significance) = normal variate i.e. 1.96
p = prevalence i.e. 87.6% = 0.876, Prevalence i.e. 87.6% was taken from a study conducted by Pollán M, et al.^4^
e = Allowable error i.e. 0.05
n = sample size

$$\begin{array}{ll} \text{Sample size (n)} & \text{= }{\text{Z}}^{\text{2}}\text{p(1}-\text{p)}/{\text{d}}^{\text{2}}\\ & \text{= }{\left(1.96\right)}^{\text{2}}\times 0.876\times (1-0.876)/{(\text{0.05)}}^{\text{2}}\\ & \text{= }166.9 \end{array}$$

Calculated sample size was 167. There were 258 COVID-19 positive cases admission from 13th April to 13th August 2020. Out of these cases 57 cases were excluded as they did not have respiratory symptoms but were admitted for other medical conditions. Out of these 57 cases, 27 were admitted for dialysis and non-respiratory medical conditions, 21 were asymptomatic who were admitted during initial days of COVID-19 and 9 patients were admitted for surgery. So, 201 symptomatic patients were analyzed in this study.

Data entry was done in excel sheet and presenting symptoms of COVID-19 positive patients were analyzed along with their comorbid conditions. The descriptive statistical analysis was done.

## RESULTS

**Figure 1:**
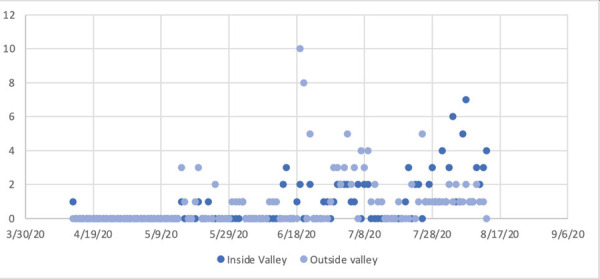
Number of patients travelling into Kathmandu valley (n=109) vs residents (n=92) of the valley in different months.

Mean age of this study population was 37.9 years (median 37) with minimum age of 2 months and maximum age of 83 years. There were 114 (56.7%) male and 87 (43.3%) female; 109 (54.2%) patients were from outside the and 92 (45.8%) were from inside of Kathmandu Valley which is the city where this hospital is situated. The proportion of patients travelling into Kathmandu valley was high during the month of June 2020, however as cases started to rise, hospital was filled with residents of Kathmandu valley (Figure 1). The most common presenting symptom on arrival to emergency department was Fever 131 (65.2%) (Table 1).

**Table 1:** Frequency of presenting symptoms on arrival at emergency (n=201)

Symptoms	Frequency (%)
Fever	131 (65.2)
Weakness	40 (19.9)
Cough	39 (19.4)
Running Nose	34 (16.9)
Shortness of breath	29 (14.4)
Headache	27 (13.4)
Sore Throat	13 (6.5)
Nausea - Vomiting	12 (6.0)
Diarrhea	11 (5.5)
Myalgia	10 (5.0)
Irritability	2 (1.0)

**Figure 2:**
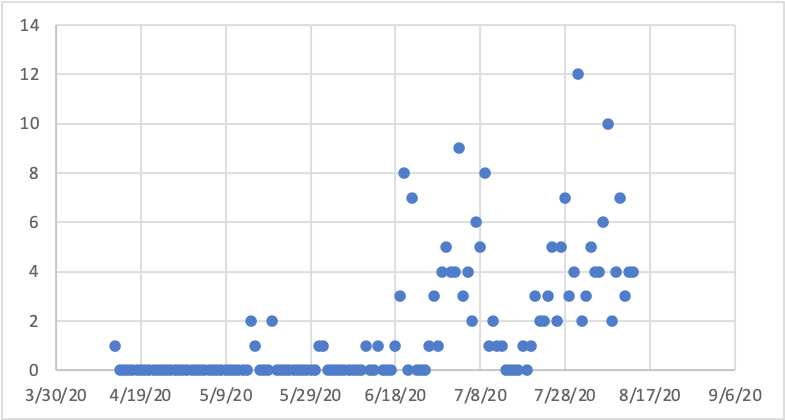
Number of symptomatic patients in timeline (n=201).

**Figure 3:**
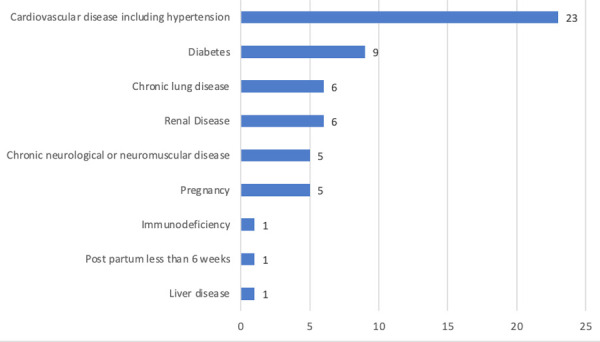
Comorbid conditions in SARS COV2 positive, symptomatic patients (n=201).

One hundred and five patients (52.2%) had single symptoms, 39 (19.4%) had two symptoms, 28 (13.9%) had three symptoms, 10 (5%) had four and five symptoms each. The symptomatic patient started to increase form the month of July 2020, (Figure 2). The most common comorbid condition was cardiovascular disease including hypertension 23 (11.4%) followed by diabetes 9 (4.5%) (Figure 3).

Shortness of breath was observed in all patient with lung disease six (100%), seven (77.7%) patient with diabetes 11 (47.8%) patient with cardiovascular condition including hypertension and two (40%) patient with chronic neurological condition.

## DISCUSSION

In our study, fever was the most common (65.2%), presenting symptoms of patients coming to COVID-19 emergency. An epidemiological review published from china also suggests fever (98.6%) as one of the common symptoms.^5^ Therefore, fever is in one of the essential criteria in the definition of suspected case.^6^ It is well known since decades that, during infection fever is caused by endogenous and exogenous pyrogens. Exogenous pyrogens are released by organism which further trigger release of endogenous pyrogens called cytokinins, which are interleukin 1,2,6 (IL1, IL2, IL6), Tissue Necrotic Factor (TNF) and Interferon alpha (INF).^7^ This cytokinins thereby acts on hypothalamus to elevation of the hypythalamic set point by increasing prostaglandin E2 (PGE2).^8^ Fever in COVID-19 is also due to similar mechanism causing release of cytokines secondary to tissue damage or hypoxia. Fever, however does not specify upper or lower respiratory tissue damage, extent of damage nor the extent of hypoxia. However, persistence of fever might be the only clue to the possibility of subtle hypoxia for a evaluating emergency physicians.

The patient with SARS-COV2 infection however, do not present immediately with shortness of breath. As seen in our study 14.4% of patient presented with clinically evident shortness of breath. Initial hypoxia can be missed as this patient are in silent hypoxia, called 'happy hypoxia'. There is a disconnection between patient's sensation of shortness of breath and hypoxaemia as evident by a study done by Tobin et al who demonstrated hypoxaemia without increase in alveolar ventilation in patients with COVID19.^9^ The signmoid shaped oxyhemoglobin curve shifts to left due to hypoxaemia driven tachypnea and and hyperpnea without obvious dyspnea. This shift causes increase in affinity of hemoglobin for oxygen thereby increasing oxygen saturation for given PaO2.^10^ Furthermore, fatigueless can also be hypothesized as a result of subtle tissue hypoxia. Weakness or fatigue was seen in 19.9% of cases which even progressed to chronic fatigue. However there was no relationship was seen between inflammatory parameter and fatigue. Moreover, 85% of subjects had normal CRP and IL-g levels.^11^

Cough was present in 19.4% of our cases. Most of the patient (52.2%) presented with single symptoms, however rest presented with combination of multiple symptoms. A meta-analysis study of COVID-19 patients, showed fever (88.8%) as the most common symptom, followed by dry cough (68%) and fatigue (33%).^12^ In similar other studies, the most common symptoms being fever, malaise and dry cough respectively and the spectrum of disease ranged from asymptomatic to septic shock and multiorgan dysfunction.^13^ Respiratory failure and respiratory distress syndrome was the leading cause of mortality in COVID19 which is triggered by a fulminant hypercytokinaemia.^14^ In adults viral infections triggers hypercytokinaemia in 3.7-4.3% of cases^15^, the cardinal feature of which is persistent fever, cytopenia and hyperferritinaeia.^16^ Therefore it is very important to understand the combination of symptoms and what it means while assessing patient in the emergency. We did not find any studies interpreting the symptom and combination of the symptom, but it is worth hypothesizing that persistence of fever and cough means involvement of lower respiratory tract while, anosmia, rhinorrhea, sore throat means involvement of upper respiratory tract. Progression of disease depends on comorbid conditions and level of symptoms with respect to day of infection.

It has been observed that patients with comorbidities have higher case fatality rate compared to those without comorbidities.^13,17^ In our study, most of the COVID19 patient admitted from emergency had cardiovascular condition, specifically hypertension. A meta-analysis study on COVID-19 comorbidities, highlighted the most comorbidities identified in these patients were hypertension (15.8%), cardiovascular and cerebrovascular conditions (11.7%), diabetes (9.4%); less common comorbidities were coexisting infection with HIV and hepatitis B (1.5%), malignancy (1.5%), respiratory illnesses (1.4%), renal disorders (0.8%), and immunodeficiency (0.01%).^12^

So, we analyzed underlying conditions of the patient dividing the comorbid conditions into three types: normal, comorbidity other than lung condition and patient with underlying lung condition. Out of these three category 100% of patient with underlying lung condition presented with shortness of breath, 40-77.7% who presented with shortness of breath various medical condition except respiratory. None of the patient without comorbid condition presented with shortness of breath. One of the published report suggested that patients with moderate to severe asthma are at a disadvantage because this virus affects their respiratory tracts, leading to increased asthmatic attacks, pneumonia, and acute respiratory distress.^18^

Shortness of breath is subtle when there is ventilation perfusion (V/Q) mismatch, however it becomes more prominent as shunt increases.^19^ It seems that progression from V/Q mismatch to shunting is quicker in patient with underlying respiratory illness in comparison to patient with non-respiratory comorbidity and then to patient without comorbid condition. So, identifying the patient hypoxia during V/Q mismatch phases and anticipating its progression is a very important skill in the emergency room.

Beside identifying, the subtle clinical signs, it is important to monitor the pattern of disease in as it directly affects the patient management in terms of resources and preparedness. During this study period, the numbers of cases in the Kathmandu valley was rising significantly as evident in figure 2 and the local cases were also rising as evident from figure 1. This is a warning sign for increase in the number of cases but not necessarily the mortality. It has been observed that mortality in COVID19 increases with age.^20^ The mean age of the patient's in our study was 37.9 years, the shift in mean age to more than 65 years is another important warning sign indicating the number of mortality irrespective of population size affected.

Symptoms of COVID-19 may appear anytime from 2 to 14 days after exposure.^18^ Patients had mean of 3.5 days from the onset of symptoms to admission in the hospital, but this varied according to age. The same study also showed that fever was present in 87.5% of patients, which persisted for 6.5 days.^21^ Acute respiratory distress syndrome was observed to have developed within 8-12 days.^22^ Therefore, it is crucial for emergency physician to observe and understand subtle clinical signs with respect to its onset and resolution time as we may not get another opportunity to save the particular patient. Timely intervention also helps in proper utilization of the resources.

## CONCLUSIONS

Fever was the most common symptoms of the patient presenting to COVID19 emergency of our hospital. Moreover, fever needs to be analyzed carefully in terms of its onset total duration and associated cough and underlying comorbid condition. The subtle signs of hypoxia can be recognized if symptoms are evaluated meticulously on presentation to emergency.
